# Cytomegalovirus antigenemia in patients with autoimmune and non-autoimmune diseases in Beijing: A 10−year single hospital experience

**DOI:** 10.1371/journal.pone.0221793

**Published:** 2019-08-28

**Authors:** Jingtao Cui, Wenjuan Yan, Hongjie Xie, Shaoxia Xu, Qiaofeng Wang, Weihong Zhang, Anping Ni

**Affiliations:** Department of Clinical Laboratories, Peking Union Medical College Hospital, Chinese Academy of Medical Sciences and Peking Union Medical College, Beijing, China; Texas Woman's University, UNITED STATES

## Abstract

**Background:**

Primary cytomegalovirus (CMV) infection is prevalent worldwide and usually results in latency in immunocompetent populations. Reactivation of latent CMV can cause life-threatening complications in immunocompromised hosts.

**Methods:**

We used the CMV Brite assay to test CMV antigenemia (pp65) in whole blood samples from 22,192 patients with or without autoimmune diseases in Beijing during 2008–2018.

**Results:**

The overall prevalence of CMV antigenemia was 19.5% (9.7%, males; 26.0%, females). The prevalence of CMV antigenemia was 35.1%, 58.6% and 11.4% in whole patients with autoimmune diseases, in patients with systemic lupus erythematosus (SLE) and in patients with non-SLE autoimmune diseases, respectively. All patients with non-autoimmune diseases, patients with HIV/AIDS or transplantation were found to have 5.0%, 27% or 14.8%, respectively. Patients≤20 years with SLE had a significantly higher prevalence of CMV antigenemia than did all SLE patients, on average. Patients>51 years with non-SLE autoimmune diseases had a significantly higher prevalence than did all patients with non-SLE autoimmune diseases, on average. The prevalence of CMV antigenemia in patients admitted to intensive-care units (ICUs) were 9.2%, which was significantly higher than that among all patients with non-autoimmune diseases. Patients with SLE had 23.8% of negative conversion of CMV antigenemia, significantly lower than the percentage of patients with non-SLE autoimmune (64.3%) and non-autoimmune (61.0%) diseases. The mean number of days to negative conversion of CMV antigenemia in patients with SLE was 35.3±35.8 days, which was significantly longer than that in patients with non-SLE autoimmune diseases (15.4±11.9 days) and non-autoimmune diseases (13.6±7.7 days).

**Conclusions:**

CMV antigenemia is found more likely in women than in men, more prevalently in patients with SLE than those with HIV/AIDS or transplant recipients, more frequently in patients admitted to ICUs. Patients with SLE had prolonged CMV antigenemia. The role of CMV appears important in SLE.

## Introduction

Human cytomegalovirus (CMV) is a member of the subfamily *Betatheherpesviridae* of the family *Herpesviridae*. CMV infection in humans has a worldwide distribution, with seroprevalence varying from 45% to 100% in different geographic regions [1] The overall CMV seroprevalence in the adult population of Germany is 56.7% [2], and that among individuals aged 15–49 years in France is 41.9% [3]. The CMV seroprevalence is 96%–98% in adult populations of mainland China [4, 5], and 91.7% among blood donors in Taiwan [6]. Most primary CMV infections are usually asymptomatic and acquired by direct, close contact with CMV-infected body fluids. If present, symptoms include infectious mononucleosis, fever, malaise, lymphocytosis and mild hepatitis. Primary CMV infection results in persistent or latent infection in immunocompetent hosts, with sites of latent infection are located in endothelial cells, peripheral blood mononuclear cells [7], and cells of the myeloid lineage are also an important site of carriage of HCMV [8]. Reactivation of CMV from latency can cause life-threatening complications if immunocompetent individuals, such as patients with AIDS, transplant recipients, patients admitted to intensive-care units (ICUs), or those with autoimmune diseases, become immunocompromised [9]. An autoimmune disease is an illness that occurs when the immune system is dysregulated and the body’s tissues are attacked by its own immune system, such as in systemic lupus erythematosus (SLE). Corticosteroids and other immunosuppressive medications are usually prescribed to reduce the diseases activity. Reactivation of CMV in immunocompromised patients may be more common in China than in Western countries because of the high prevalence of previous CMV infection in China.

The CMV antigenemia test was first described in 1988 [10]. The test is a sensitive, specific and rapid assay for the early diagnosis of CMV infection, including reactivation of latent CMV, on the basis of detection of the 65-kDa lower matrix phosphoprotein (pp65) in the nuclei of the peripheral blood leukocytes using immunocytochemical or immunofluorescence techniques. The CMV antigenemia assay was introduced in our laboratory in the early 2000s to monitor active CMV infection in patients with HIV/AIDS and in transplantation patients during immunosuppressive chemotherapy. Since then, a considerable number of blood samples from patients with autoimmune diseases have been sent to our laboratory for testing. The CMV antigenemia assay has been found to be useful for the diagnosis of active CMV infection in patients with autoimmune diseases in the several studies [11–14], with one study in our hospital in 2014 analyzing 105 patients with SLE [14]. The CMV antigenemia assay has a good correlation with CMV DNAemia detected using polymerase chain reaction (PCR), although PCR may be more sensitive in low levels of viremia [15–16]. In this study, we aimed to analyze the results of the CMV antigenemia assay in patients with autoimmune diseases and those with non-autoimmune diseases using a large sample size and 10-year time span, from 2008 to 2018.

## Methods

### Ethics statement

The study was approved by the Ethics Committee (reference no. S-K528) of Peking Union Medical College Hospital (PUMCH). The requirement for informed consent was waived as the blood samples used were collected and tested for routine medical purpose, and we only analyzed the results of the CMV antigenemia assay, which had been reported to the patients and to physicians. Patients’ identifying information was removed before the analysis.

### Study samples

A total of 22,192 whole blood samples were collected from patients visiting the PUMCH from May, 2008 to December, 2018; all samples were subsequently tested for CMV antigenemia. All patient categories and demographic information were based on our hospital data. In general, whether patients were in a disease state was uncertain.

The inclusion criteria were blood samples from patients who were Chinese, had complete demographic information, and had laboratory results for the CMV antigenemia test.

Exclusion criteria were blood samples from patients who: were foreigners (as the study was designed for Chinese patients only), from those who had no demographic information, or from those had no laboratory test results for CMV antigenemia. More than one of blood samples collected in fewer than 3 days from one patient were considered repeated samples, and only the first sample was included for analysis; the remaining samples were excluded.

### Laboratory testing

Peripheral whole blood samples were collected using sterile vacuum blood collection tubes contain EDTA.K2 anticoagulant. The CMV Brite assay (IQ products BV, Groningen, the Netherland) was performed to test for CMV antigenemia according to the manufacturer’s instructions. Briefly [17], peripheral blood leukocytes in whole blood samples were separated with dextran and erythrocytes were lysed with ammonium chloride lysis buffer. The leukocytes were counted using a hemocytometer and about 1.5×10^5^/0.1mL cells were applied to each of two slides through cytocentrifugation (Cytospin 4, Shandon, Thermo Scientific, UK) at 1000rpm for 5 minutes. Cells on the slides were fixed with formaldehyde, permeabilized with Igepal CA-630, and stained with anti-CMV pp65 antibodies (C10/C11, IgG1), and re-stained with FITC-labeled rabbit anti-mouse IgG conjugate. For each assay, positive and negative control slides from Brite kit was treated using a similar procedure as that above. Finally, the slides were read under a fluorescence microscope (Olympus BX51, Tokyo, Japan). Polylobate perinuclear yellow-green fluorescent staining of leukocytes was used to determining positive CMV antigenemia. The following cut-off values were used to determine positive CMV antigenemia: positive, one or more CMV antigen-positive cells present per duplicate stain; and negative, no CMV antigen-positive cells present per duplicate stain.

### Statistical analysis

Our laboratory information system (LIS) software was installed on a server. Results of CMV antigenemia assays were stored in the server after testing was completed and the results were reported. To protect patients’ identifying information, the LIS was only accessible to staff of the virology division using passwords. The data were exported to Microsoft Excel 2007 (Microsoft Corp., New York, NY, USA), where they were sorted and the results were preliminarily calculated. Statistical analysis was performed using IBM SPSS software version 21.0 (IBM Corp., Armonk, NY, USA). *P*-values <0.05 were considered statistically significant. The chi-squared test with continuity correction and an independent samples *t*-test were used to compare the prevalence of CMV antigenemia and the mean number of days until negative conversion of CMV antigenemia.

## Results

### Prevalence of CMV antigenemia

From 2008 to 2018, a total of 22,192 whole blood specimens were tested for CMV antigenemia. The general information of patients with autoimmune diseases and non-autoimmune diseases is shown in Table 1. There were 10,749 (48.4%) patients with autoimmune diseases and 11,443 (51.6%) with non-autoimmune diseases. Among the 10,749 patients with autoimmune diseases, 5,379 (50.0%) had SLE and 5,370 (50.0%) had non-SLE autoimmune diseases. The age range of the total 22,192 patients was from 1 day to 97 years old, and the mean age was 41.6±19.4 years old with a median of 41 years. For patients with SLE, the mean age was 31.4±14.8 years with a median of 28 years.

**Table 1:** General information of patients with autoimmune and non-autoimmune diseases tested for cytomegalovirus antigenemia at Peking Union Medical College Hospital, Beijing (2008 to 2018).

	Mean age (y)	Male n (%)	Female n (%)	Total samples.
Autoimmune diseases				
SLE	31.4±14.8	660(12.3)	4,719(87.7)	5,379
Non-SLE				
Still disease and AOSD	31.5±13.8	45(20.0)	179(80.0)	224
Rheumatoid arthritis	49.8±18.9	69(23.5)	224(76.5)	293
Sjogren syndrome	51.0±13.4	28(8.2)	315(91.8)	343
Polymyositis and dermatomyositis	46.4±15.8	222(35.6)	402(64.4)	624
Vasculitis	47.1±19.3	414(52.0)	382(48.0)	796
Inflammatory bowel disease	39.6±15.2	778(60.0)	520(40.0)	1,298
Other or undefined autoimmune diseases	41.9±19.3	672(37.5)	1,120(62.5)	1,792
Subtotal of autoimmune diseases	37.5±17.5	2,888(26.9)	7,861(73.1)	10,749
Non-autoimmune diseases				
Cushing syndrome (ACTH-related)	48.3±18.5	15(30.6)	34(69.4)	49
HLH	38.6±19.1	21(37.5)	35(62.5)	56
HIV/AIDS	41.2±10.9	99(89.2)	12(10.8)	111
Post-transplantation#	42.2±14.5	131(60.6)	85(39.4)	216
Elevated serum liver enzymes	38.7±20.9	163(45.2)	198(54.8)	361
Haematologic-/oncologic diseases##	47.0±15.9	552(61.2)	350(38.8)	902
Pneumonia	57.7±19.1	716(54.6)	595(45.4)	1,311
FUO	40.4±19.3	1,412(44.0)	1,794(56.0)	3,206
Other or undefined non-autoimmune diseases	46.5±20.7	2,776(53.1)	2,455(46.9)	5,231
Subtotal of non-autoimmune diseases	45.7±20.2	5,885(51.4)	5,558(48.6)	11,443
Total	41.6±19.4	8,773(39.5)	13,419(60.5)	22,192

CMV, cytomegalovirus; SLE, systemic lupus erythematosus; AOSD, adult-onset Still disease; ACTH, adenocorticotropic hormone; HLH, hemophagocytic lymphohistiocytosis; FUO, fever of unknown origin

# Post-transplantation, includes kidney transplantation (101 cases), liver transplantation (3 cases) and allogeneic stem cell transplantation (112 cases)

## Hemato—/oncologic diseases, include myelodysplastic syndrome (49 cases), POEMS syndrome (110 cases), multiple myeloma (91 cases), lymphoma (359 cases) and leukemia (293 cases)

Note: % values indicate the proportion of men or women in each disease category, this differs from the meaning in Table 2.

As shown in Table 2, the overall prevalence of CMV antigenemia was 19.5% (4,335/22,192); 9.7% (847/8,773) for male patients and 26.0% (3,488/13,419) for female patients, The total sex difference was significant (**χ**^*2*^ = 899.9, *P*<0.001). The average prevalence of CMV antigenemia in patients with autoimmune diseases was 35.1% (3,768/10,749), 58.6% (3,154/5,379) in patients with SLE and 11.4% (614/5,370) in patients with non-SLE autoimmune diseases, the latter (11.4%) was significantly higher than 5.0% (567/11,443), the average prevalence in patients with non-autoimmune diseases (**χ**^*2*^ = 227.1, *P*<0.001). Sex differences in the prevalence of CMV antigenemia were not significant between male patients and female patients with SLE (57.7% vs. 58.8%, **χ**^*2*^ = 0.215, *P* = 0.643).

**Table 2:** Prevalence of cytomegalovirus antigenemia in male and female patients with autoimmune diseases and non-autoimmune diseases at Peking Union Medical College Hospital, Beijing (2008 to 2018).

	Male CMV antigenemia n (%)	Female CMV antigenemia n (%)	Total CMV antigenemia n (%)	χ^*2*^	*P*-value
Autoimmune diseases					
SLE	381(57.7)	2,773(58.8)	3,154(58.6)	**0.215**	**0.643###**
Non-SLE					
Still disease and AOSD	3(6.7)	13(7.3)	16(7.1)		
Rheumatoid arthritis	14(20.3)	44(19.6)	58(19.8)		
Sjogren syndrome	4(14.3)	67(21.3)	71(20.7)		
Polymyositis and dermatomyositis	36(16.2)	56(13.9)	92(14.7)	**0.427**	**0.514**
Vasculitis	30(7.2)	39(10.2)	69(8.7)	**1.845**	**0.174**
Inflammatory bowel disease	58(7.5)	37(7.1)	95(7.3)		
Subtotal of six autoimmune diseases above	145(9.3)	256(12.7)	401(11.2)	**9.54**	**0.002**
Other or undefined autoimmune diseases	59(8.8)	154(13.8)	213(11.9)	**9.438**	**0.002**
Subtotal of autoimmune diseases	585(20.3)	3,183(40.5)	3,768(35.1)	**378.96**	**0.000**
Non-autoimmune diseases					
Cushing syndrome (ACTH-related)	0(0)	2(5.9)	2(4.1)		
HLH	6(28.6)	12(34.3)	18(32.1)		
HIV/AIDS	26(26.3)	4(33.3)	30(27.0)		
Post-transplantation#	23(17.6)	9(10.6)	32(14.8)	**1.47**	**0.225**
Elevated serum liver enzymes	7(4.3)	9(4.5)	16(4.4)		
Haematologic-/oncologic diseases##	30(5.4)	16(4.6)	46(5.1)		
Pneumonia	62(8.7)	62(10.4)	124(9.6)		
FUO	24(1.7)	71(4.0)	95(3.0)	**12.04**	**0.001**
Other or undefined non-autoimmune diseases	84(3.0)	120(4.9)	204(3.9)	**11.56**	**0.001**
Subtotal of non-autoimmune diseases	262(4.5)	305(5.5)	567(5.0)	**6.29**	**0.012**
Total	847(9.7)	3,488(26.0)	4,335(19.5)	**899.9**	**0.000**

CMV, cytomegalovirus; SLE, systemic lupus erythematosus; AOSD, adult-onset Still disease; ACTH, adenocorticotropic hormone; HLH, hemophagocytic lymphohistiocytosis; FUO, fever of unknown origin

# Post-transplantation, includes kidney transplantation (101 cases), liver transplantation (3 cases), allogeneic stem cell transplantation (112 cases)

## Hematologic-/oncologic diseases, include myelodysplastic syndrome (49 cases), POEMS syndrome (110 cases), multiple myeloma (91 cases), lymphoma (359 cases) and leukemia (293 cases)

### Comparison of prevalence of cytomegalovirus antigenemia between male and female patients with SLE. Same patterns were used for other comparisons in patients with other diseases or symptoms in Table 2.

Note: % values indicate positive rate of CMV antigenemia in men or women, or in both men and women, in each disease category, this differs from the meaning in Table 1.

### Age-related prevalence of CMV antigenemia

Details of age-related prevalence of CMV antigenemia in patients with autoimmune diseases and non-autoimmune diseases are shown in Table 3. For patients with SLE <20 years old, the prevalence of CMV antigenmia was between 63% (<10yrs) and 67% (11–20yrs); both significantly higher than the 58.6% (3,154/5,379) in all SLE patients on average. In contrast, for patients with non-SLE autoimmune diseases >51 years old, the prevalence of CMV antigenemia was between 14% (≥61yrs) to 15% (51–60yrs); both significantly higher than 11.4% (614/5,370) in all patients with non-SLE autoimmune diseases on average.

**Table 3:** Age-related prevalence of cytomegalovirus antigenemia in patients with SLE, non-SLE autoimmune diseases and non-autoimmune diseases at Peking Union Medical College Hospital, Beijing (2008 to 2018).

	SLE CMV antigenemia Positive, n (%)			Non-SLE autoimmune CMV antigenemia Positive, n (%)	Non-autoimmune CMV antigenemia Positive, n (%)	χ^*2*^	*P*-value
	Male	Female	Total				
0–10y	9(39.1)	144(70.2)	**153(67.1)**	15(9.0)	12(3.3)	**48.93**	**0.000#**
11–20y	112(67.9)	628(62.6)	**740(63.4)**	31(6.1)	31(3.4)	**8.68**	**0.003##**
21–30y	99(57.2)	807(57.8)	906(57.7)	67(8.2)	94(5.0)		
31–40y	70(53.8)	463(56.3)	533(56.0)	72(9.2)	62(3.8)		
41–50y	55(58.5)	411(57.3)	466(57.5)	116(11.3)	85(5.1)		
51–60y	5(20.8)	220(54.9)	225(53.0)	***167(15*.*5)***	99(5.1)	**13.51**	**0.000###**
61y–	31(60.8)	100(57.5)	131(58.2)	***146(14*.*6)***	184(6.0)	**7.88**	**0.005####**
Total	381(57.7)	2,773(58.8)	**3,154(58.6)**	***614(11*.*4)***	567(5.0)		

Autoimmune, autoimmune diseases

**#** Comparison of the prevalence of cytomegalovirus antigenemia between SLE patients ≤10 years old (63.4%, 740/1,168) and total SLE patients, on average (58.6%, 3,154/5,379).

**##** Comparison of the prevalence of cytomegalovirus antigenemia between SLE patients aged 11–20 years (67.1%, 153/228) and total SLE patients, on average (58.6%, 3,154/5,379).

**###** Comparison of prevalence of cytomegalovirus antigenemia between patients with non-SLE autoimmune diseases aged 51–60 years (67.1%, 153/228) and all patients with non-SLE autoimmune diseases (11.4, 614/5,370).

**####** Comparison of prevalence of cytomegalovirus antigenemia between patients with non-SLE autoimmune diseases >60 years old (14.6%, 146/998) and all patients with non-SLE autoimmune diseases (11.4, 614/5,370).

### Prevalence of CMV antigenemia among patients in different hospital departments

Table 4 shows the prevalence of CMV antigenemia among patients with non-autoimmune diseases in various hospital departments. The prevalence of CMV antigenemia in patients admitted to ICUs (including internal medicine ICU, surgical ICU and coronary care unit) was 9.2% (122/1328), this was significantly higher than the prevalence of 5.0% (567/11,443) among all patients with non-autoimmune diseases on average (**χ**^*2*^ = 40.9, *P*<0.001).

**Table 4:** Prevalence of cytomegalovirus antigenemia in patients with non-autoimmune diseases in various departments of Peking Union Medical College Hospital, Beijing (2008 to 2018).

Department	Total samples	CMV antigenemia positive(n)	%	*χ*^*2*^	*P*-value
Pediatrics	486	12	2.5		
Internal Medicine	4,060	172	4.2		
Immunology	657	49	7.5	**7.548**	**0.006##**
Infectious Diseases	2,073	65	3.1		
Emergency	1,313	78	5.9		
ICUs#	1328	122	9.2	**40.9**	**0.000**
Other	1,526	69	4.5		
Total	11,443	567	5.0		

# ICUs (intensive care units) include internal medicine ICU (1024 cases), surgical ICU (258 cases) and coronary care unit (46 cases)

## Comparison of the incidence of cytomegalovirus antigenemia among patients admitted to the department of immunology (7.5%, 49/657) and patients admitted to all departments, on average (5.0%, 567/11,443). The same patterns were used for other comparisons among patients in Table 4.

### Negative conversion of CMV antigenemia

A total of 2,481 (56.5%, 2,481/4,335) blood samples with CMV antigenemia previously were recollected and retested after 3 to 2,884 days (up to 7.9 years) from the time of the previous assay. The 2,481 patients with positive CMV antigenemia who were being followed up by analyzing subsequent blood sample results were from the same patients. Patients with SLE had the lowest proportion (23.8%, 461/1,936) of negative conversion of CMV antigenemia, as compared with patients with non-SLE autoimmune diseases (64.3%, 202/314, *χ*^*2*^ = 211.5, *P*<0.001) and those with non-autoimmune diseases (61.0%, 141/231, *χ*^*2*^ = 140.7, *P*<0.001). A comparison of proportion of negative conversion of CMV antigenemia among patients is shown in Table 5. The sex difference was significant between male and female patients with non-SLE autoimmune diseases and non-autoimmune diseases (*χ*^*2*^ = 8.12, *P* = 0.004), but this was not significant between male and female patients with SLE (*χ*^*2*^ = 0.03, *P* = 0.862).

**Table 5:** Proportions of negative conversion of cytomegalovirus antigenemia among male and female patients with autoimmune diseases and non-autoimmune diseases at Peking Union Medical College Hospital, Beijing (2008 to 2018).

	Male CMV antigenemia Neg conv, n (%)	Female CMV antigenemia Neg conv, n (%)	Total CMV antigenemia Neg conv, n (%)	*χ*^*2*^	*P*-value
SLE	58(24.5)	403(23.7)	461(23.8)	**0.03**	**0.862#**
Non-SLE	88(75.9)	114(57.6)	202(64.3)	**8.12**	**0.004##**
Non-autoimmune diseases	68(64.2)	73(58.4)	141(61.0)		
Total	214(46.6)	590(29.2)	804(32.4)	**51.2**	**0.000###**

Neg conv, negative conversion

# Comparison of proportions of negative conversion of cytomegalovirus antigenemia between male and female patients with SLE.

## Comparison of proportions of negative conversion of cytomegalovirus antigenemia between male and female patients with non-SLE and non-autoimmune diseases.

###Comparison of proportions of negative conversion of cytomegalovirus antigenemia between all male and all female patients.

### Days until negative conversion of CMV antigenemia

The number of days from positive to negative conversion of CMV antigenemia in patients with SLE ranged from 3 to 2,884 days, and the mean ± standard deviation (SD) was 133.9±419.9 days with a median of 21 days. Clearly, 2,884 days (7.9 years) is meaningless for calculating the time to negative conversion of CMV antigenemia as there might be many rounds of disease activities during such a long period. Hence, we established the definition that data of 10% of patients with the longest periods to negative conversion would not be used in the calculation and this definition was also applied to calculations for patients with non-SLE autoimmune diseases and non-autoimmune diseases. The number of days was calculated according to above definition and the results are shown in Table 6. The mean number of days to negative conversion of CMV antigenemia in patients with SLE were 35.3±35.8 days, which was significantly more than the 15.4±11.9 days in patients with non-SLE autoimmune diseases (*t* = 7.32, *P*<0.001) and the 13.6±7.7 days in patients with non-autoimmune diseases (*t* = 6.76, *P*<0.001). The mean number of days until negative conversion of CMV antigenemia was significantly different between male and female patients with non-SLE autoimmune diseases and non-autoimmune diseases (*t* = 2.80, *P* = 0.005) but not between male and female patients with SLE (*t* = 0.287, *P* = 0.774).

**Table 6:** Comparison of the number days until negative conversion of cytomegalovirus antigenemia in male and female patients with autoimmune diseases and non-autoimmune diseases at Peking Union Medical College Hospital, Beijing (2008 to 2018).

	Male Number of days (mean±SD)	Female Number of days (mean±SD)	Total Number of days (mean±SD)	*t*	*P-* value
SLE	36.7±37.2	35.1±35.8	35.3±35.8	**0.287**	**0.774#**
Non-SLE	13.6±8.2	17.1±15.3	15.4±11.9	**2.80**	**0.005##**
Non-autoimmune diseases	12.1±6.5	15.6±10.2	13.6±7.7		
Total	17.0±13.9	28.7±29.8	25.4±26.2	**4.00**	**0.000###**

# Comparison for number of days from positive to negative conversion of CMV antigenemia between male and female patients with SLE.

## Comparison for number of days from positive to negative conversion of CMV antigenemia between male and female patients with non-SLE and non-autoimmune diseases.

### Comparison for number of days from positive to negative conversion of CMV antigenemia between all male and all female patients.

## Discussion

As shown in Table 2, the average prevalence (35.1%) of CMV antigenemia in patients with autoimmune diseases has exceeded the prevalence in traditionally immunocompromised patients, such as 27.0% in patients with HIV/AIDS and 14.8% in patients post-transplantation. Among our patients with autoimmune diseases, the prevalence of CMV antigenemia in those with SLE (58.6%) was much higher than in patients with non-SLE autoimmune diseases: for example, 20.7% in patients with Sjogren syndrome, 19.8% in patients with rheumatoid arthritis and 14.7% in patients with polymyositis and dermatomyositis. There were many studies supporting the hypothesis that CMV infection plays a role in inducing or triggering autoimmune diseases such as SLE [18–22]. In this study, the 58.6% of CMV antigenemia in patients with SLE supports this hypothesis, and reminds that the association between CMV and SLE is much closer than the association between CMV and non-SLE autoimmune diseases. Unfortunately, reactivation of latent CMV in patients with autoimmune diseases due to immunosuppressive drugs will be more common in China than in developed countries because of the higher prevalence of primary CMV infection, which means a higher prevalence of CMV latency in China. It has been shown in our previous research [23] as well as other studies [24–26] that Epstein–Barr virus (EBV), another herpesvirus, is also more closely associated with SLE than with non-SLE autoimmune diseases; this indicates that both CMV and EBV are more involved in SLE than in non-SLE autoimmune diseases [27–29].

For non-autoimmune diseases, active CMV infection is also common in patients with immunocompromised conditions or those taking immunosuppressive medications containing corticosteroids, such as patients with HIV/AIDS or hemophagocytic lymphohistiocytosis [30] and patients post transplantation [31]. In our study, as shown in Table 2, excessive endogenous corticosteroid in patients with ACTH-related Cushing syndrome did not seem to trigger CMV reactivation, although there is a previous report of opportunistic CMV infection occurring in a patient with ACTH-related Cushing syndrome [32]. In our study, there were 49 cases of ACTH-related Cushing syndrome and 4.1% (2/49) of CMV antigenemia among them, with one patient (a 50-year-old female) testing positive twice for CMV antigenemia. That patient had Cushing syndrome as well as septic shock and acute respiratory distress syndrome; hence, she was admitted to the ICU once. Therefore, the administration of exogenous corticosteroids during medical treatment could not be excluded. We presume that low levels of chronic and autonomous secretion of cortisol in patients with ACTH-related Cushing syndrome may have less effect on dysregulation of the immune system than high dosage of exogenous corticosteroids given as immunosuppressant drugs to treat patients with autoimmune diseases, such as SLE.

As shown in Table 2, whole female patients with non-SLE autoimmune diseases had significantly higher prevalence of CMV antigenemia than the male patients although sex differences were not found in any particular disease, such as polymyositis and dermatomyositis, or vasculitis due to the limit of sample size. Similarly, females with non-autoimmune diseases were also observed to have higher prevalence of CMV antigenemia than males, confirming that women with non-SLE autoimmune or non-autoimmune diseases are more vulnerable to CMV reactivation than men.

Adolescent patients with SLE equal or less than 20 years old were more likely to have CMV antigenemia than adult or elderly patients with SLE, as shown in Table 3. The reason could be that immunosuppressive treatment is more frequently prescribed among teenagers with SLE [33, 34] because pediatric SLE is more aggressive and more often involves major organs. However, CMV infection impairs the immune profile and function during normal human aging [35–37], leaving older adults more susceptible to CMV reactivation. This could be the reason patients over 51 years of age with non-SLE autoimmune diseases were found to have higher rate of CMV antigenemia than young patients in our study (Table 3).

To compare the prevalence of CMV antigenemia among various departments (Table 4), we only analyzed data of patients with non-autoimmune diseases because the department of immunology in our hospital is responsible for most diagnosis and treatment of patients with autoimmune diseases. The prevalence of CMV antigenemia in patients admitted to the ICUs was 9.2%, the highest among all patients with non-autoimmune diseases in our study. Active CMV infection is increased among immunocompetent patients admitted to the ICU [38, 39] and this is thought to be because of reactivation from latency rather than primary infection [40]. In a systematic review, Li et al [41] found an overall rate of 27% CMV infection and 31% of CMV reactivation among immunocompetent patients in the ICU, owing to the following reasons: being an elderly patient, having undergone surgery and received massive transfusion, and having received corticosteroids or catecholamines. The next highest rate (7.5%) of CMV antigenemia in our study was seen in patients among the department of immunology; we suppose that the diagnosis of autoimmune diseases (a higher prevalence of CMV antigenemia is expected) had not be established in some patients among department of immunology when CMV antigenemia was tested.

Zhang, et al [14] reported that the proportions of negative conversion of CMV antigenemia in 105 patients with SLE reached to 54.1% after 14–21 days of inductive treatment with ganciclovir. The proportion of CMV antigenemia of negative conversion among patients with SLE in our study was only 23.8%, significantly lower than the 64.3% in patients with non-SLE autoimmune diseases and the 61.0% in patients with non-autoimmune diseases (Table 5). Meanwhile, the mean number of days until negative conversion of CMV antigenemia in patients with SLE was 35.3±35.8 days, which was much longer than the mean 15.4±11.9 days in patients with non-SLE autoimmune diseases and the mean 13.6±7.7 days in patients with non-autoimmune diseases (Table 6). Interestingly, different from male patients with non-SLE autoimmune or non-autoimmune diseases, male patients with SLE did not have a lower prevalence of CMV antigenemia and higher percentage and short period to negative conversion of CMV antigenemia than female patients with SLE, suggesting that the association between CMV and SLE predominate over the sex difference. Furthermore, CMV viral activity has been found to be associated with high SLE disease activity [42], high CMV viral loads are associated with a longer course of SLE disease [43] and active CMV infection is a mortality risk factor in patients with SLE [44]. There was a hypothesis raised in the review of Doaty et al [45], which we may call it “vicious cycle”, that is CMV infection plus other factors cause SLE, SLE is medicated with immunosuppressant and reactivation of CMV from latency owing to immunosuppressant exacerbates SLE eventually.

## Supporting information

S1 File Excel database of CMVpp65 antigenemia between 2008 and 2018. (XLSX)
